# EEG‐Based Multifocal Tomographic Neurofeedback in Older Individuals With Chronic Tinnitus Does Not Lead to Persistent Electrophysiological Changes

**DOI:** 10.1002/brb3.71293

**Published:** 2026-04-12

**Authors:** Stefan Elmer, David Talaska, Nicole Peter, Nathalie Giroud, Patrick Neff, Tobias Kleinjung, Martin Meyer

**Affiliations:** ^1^ Department of Computational Linguistics, Computational Neuroscience of Speech & Hearing University of Zurich Zurich Switzerland; ^2^ Competence Center Language & Medicine University of Zurich Zurich Switzerland; ^3^ Institute for the Interdisciplinary Study of Language Evolution University of Zurich Zurich Switzerland; ^4^ Department of Otorhinolaryngology University Hospital Zurich, University of Zurich Zurich Switzerland; ^5^ Center for Neuroscience Zurich University and ETH of Zurich Zurich Switzerland; ^6^ Department of Psychiatry and Psychotherapy University of Regensburg Regensburg Germany

**Keywords:** EEG, health‐related variables, neurofeedback, power spectra, psychological outcomes, tinnitus loudness and distress, source‐estimation

## Abstract

**Purpose:**

Tinnitus is typically a chronic condition characterized by phantom hearing sensations in the absence of external sound sources. Although there is currently no commonly agreed neurobiological model of tinnitus generation and chronification, several electrophysiological parameters have been proposed as potential neural markers to objectify tinnitus symptoms. Accordingly, electroencephalography‐based (EEG) neurofeedback may prove to be a promising symptom‐relieving intervention to directly influence the development, maintenance, and distress of tinnitus. In the present clinical study, we examined the effects of low‐ and high‐intensity multifocal tomographic EEG neurofeedback in two matched cohorts of older participants with chronic subjective tinnitus who learned to regulate electrical activity in three circumscribed brain regions, namely the auditory cortex, anterior insula, and dorsal anterior cingulate.

**Method:**

Using a crossover design in which the two groups received 1 or 2 weekly training sessions in the first month, with the same number of total training sessions after 2 months, participants attempted to increase the alpha/delta power ratio in the bilateral auditory cortex as well as the theta/beta ratio in the dorsal anterior cingulate cortex and the anterior insula. We evaluated the impact of the EEG‐based neurofeedback training on self‐reported tinnitus scores, psychological variables, and health‐related dimensions. The effectiveness of multifocal tomographic neurofeedback and training intensity was also assessed using resting‐state EEG data collected before and after 1 and 2 months of training, as well as 3 and 6 months after the last training session.

**Finding:**

Neurofeedback protocols were generally associated with lower tinnitus distress and had a positive impact on subjective general health status in the low‐intensity training group. However, contrary to our expectations, we did not find any neural effect of neurofeedback for the trained brain regions or on subjective tinnitus perception.

**Conclusion:**

Taken together, these results provide a critical perspective on the relationship between multifocal tomographic neurofeedback and group‐based resting‐state EEG as a marker of training‐dependent changes of neural activity.

## Graphical abstract

The effects of low‐ and high‐intensity multifocal tomographic electroencephalography‐based (EEG) neurofeedback were examined in two matched cohorts of older participants with chronic subjective tinnitus who learned to modulate electrical activity in the auditory cortex, anterior insula, and dorsal anterior cingulate. We did not find any neural effect of neurofeedback for the trained brain regions or on subjective tinnitus perception.

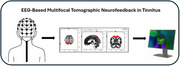

## 1 Introduction

### 1.1 Tinnitus and Available Treatments

Tinnitus commonly manifests as phantom hearing sensations, has an estimated prevalence in the range of 9–26% (McCormack et al. 2014; Sahlsten et al. 2018), and is often associated with pure‐tone hearing loss (HL) (Axelsson and Ringdahl 1989; Savastano 2008). Although this hearing‐related condition is not life‐threatening, it often leads to distress (Adjamian et al. 2014; Axelsson and Ringdahl 1989; De Ridder et al. 2021; Savastano 2008), depression, and anxiety (Dobie 2003; Langguth, Landgrebe, Kleinjung, Sand, and Hajak 2011). Such psychological states may worsen over time (Vielsmeier et al. 2020; Wallhäusser‐Franke et al. 2017) and can impair the quality of life causing frustration, anger, loss of concentration, and insomnia (Isaacson, Moyer, Schuler, and Blackall 2003; Lockwood, Salvi, and Burkard 2002; Sweetow 1986). To date, there are still no effective and permanent treatment options for tinnitus available, but only symptom‐relieving interventions such as acoustic stimulation, behavioral therapies, hearing aids, or pharmaceutical agents (Langguth et al. 2013). Nevertheless, recent approaches such as bimodal neuromodulation (Conlon et al. 2022) and fMRI‐based neurofeedback (Emmert et al. 2017) have shown encouraging results in reducing tinnitus symptoms.

### 1.2 EEG Markers of Tinnitus Thalamocortical Dysrhythmia

Although the pathophysiology of tinnitus, there is at least some progress in identifying the neural underpinnings related to the persistent and distressing nature of phantom sensations (Elmer et al. 2023; Langguth et al. 2013; Lenarz et al. 1993). Drawing on this background, the perceptual, psychological, and affective dimensions of chronic tinnitus are thought to be mediated by neural networks involved in the generation of auditory sensations, the disregulation of valence and arousal, the attribution of salience, and the maintenance of tinnitus‐related features in memory (De Ridder, Congedo, and Vanneste 2015; Langguth et al. 2013). Vivid auditory percepts are likely generated as a byproduct of synchronous hyperactivity of auditory cortical neurons (Eggermont 2007, 2008; Llinás et al. 1999; Okamoto et al. 2010), whereas tinnitus‐related valence and arousal may be mediated by extra‐auditory regions, namely the anterior cingulate cortex, the anterior insula, and the amygdala (De Ridder et al. 2006; Golm et al. 2013), as well as frontal and parietal brain regions (Elmer et al. 2023). In addition, tinnitus awareness has been proposed to depend on a salience network consisting of the anterior insula, the anterior cingulate cortex, and the thalamus (Langguth et al. 2013), while the amygdala‐hippocampal complex may be responsible for the persistence of phantom sensations through the reactivation of tinnitus‐related features from auditory memory (Berger et al. 2024; De Ridder et al. 2006).

There is reason to believe that the perceptual features of tinnitus during the generation of the phantom sound arise from a disruption of thalamocortical resonance, which leads to an imbalance between excitation and inhibition in the auditory cortex (De Ridder et al. 2015; Llinás et al. 1999). This so‐called phenomenon of thalamocortical dysrhythmia is based on the assumption that, as a result of hair cell collapse in the cochlea, the auditory thalamus generates spontaneous low‐frequency oscillations that lead to delta wave synchronization (∼ 1–3 Hz) in the auditory cortex (De Ridder, Vanneste, et al. 2015; Llinás et al. 1999; Meyer et al. 2014). As a further consequence of cochlear hair cell damage, auditory cortical neurons lose their lateral inhibition properties, leading to neural desynchronization in the alpha frequency range (∼ 8–12 Hz) (De Ridder, Vanneste, et al. 2015; Eggermont 2007; Meyer et al. 2014). Such reduced inhibitory functions are also thought to promote spontaneous gamma oscillations in the auditory cortex (> 30 Hz), which may represent one of the neurophysiological factors responsible for the salience of tinnitus sensations (De Ridder, Vanneste, et al. 2015). Importantly, the disturbance of thalamocortical resonance as a contributing mechanism to tinnitus has also been supported by several resting‐state EEG studies, which provided evidence of reduced alpha (Lorenz et al. 2009; Schlee et al. 2014; Weisz et al. 2005) and increased delta (Kahlbrock and Weisz 2008; Song, De Ridder, Schlee, Van de Heyning, and Vanneste 2013; Weisz et al. 2005) and gamma (Lorenz et al. 2009; Sedley et al. 2012; Van Der Loo et al. 2009) spectral power in participants with chronic tinnitus compared to unaffected individuals. In addition, some resting‐state EEG studies have also shown an association between tinnitus and reduced theta (∼ 4–7 Hz) and increased beta (∼ 13–30 Hz) power in the insula and anterior cingulate cortex (De Ridder, Congedo, et al. 2015; Meyer et al. 2017), although these two electrophysiological parameters were not originally part of the thalamocortical dysrhythmia model (De Ridder, Vanneste, et al. 2015; Llinás et al. 1999).

### 1.3 EEG‐related Neurofeedback Approaches for Tinnitus

Neurofeedback is a non‐invasive technique that allows individuals to gain voluntary control over specific aspects of their brain activity by providing real‐time feedback from neurophysiological signals (Emmert et al. 2017; Gninenko et al. 2024; Haller et al. 2013; Sitaram et al. 2017). In the present study, we focus exclusively on EEG‐based neurofeedback, which forms the foundation for the training protocol and the electrophysiological outcome measures assessed. Typically, the EEG signals are recorded, processed, and presented to the participants in a continuous or intermittent feedback format, such as visual or auditory cues. By reinforcing desired brain activity patterns and suppressing undesired ones, neurofeedback aims to induce neuroplastic changes that can improve cognitive, emotional, or sensory functioning. In the context of tinnitus, This approach has been explored as a method to modulate abnormal neural oscillations and reduce tinnitus‐related distress.

Since spontaneous neural activity may provide crucial information for the objectification of chronic tinnitus (Meyer et al. 2014; Meyer et al. 2017; Neff et al. 2019; Weisz et al. 2005), EEG‐based neurofeedback could represent a fruitful intervention to modulate deviant neural oscillations and thereby alleviate symptoms. Neurofeedback is based on cybernetic principles that use an organism's physiological state to regulate desired brain functions through closed signal loops and operant conditioning (Elmer and Jäncke 2014; Gunkelman and Johnstone 2005), and has already found application in several areas (Schoenberg and David 2014), such as depression (Young et al. 2014), epilepsy (Tan et al. 2009), attention‐deficit hyperactivity disorder (Maurizio et al. 2014), addiction (Dehghani‐Arani, Rostami, and Nadali 2013), and tinnitus (Güntensperger et al. 2017). Based on the framework of thalamocortical dysrhythmia (De Ridder, Vanneste, et al. 2015; Llinás et al. 1999), most tinnitus neurofeedback studies commonly used training protocols with the aim of decreasing delta and/or increasing alpha power at the sensor level, which corresponds to a change in the alpha‐delta ratio (Crocetti et al. 2011; Dohrmann et al. 2007; Guillard et al. 2021; Güntensperger et al. 2019; Hartmann et al. 2014). However, only some (Dohrmann et al. 2007; Güntensperger et al. 2019) but not all (Crocetti et al. 2011; Guillard et al. 2021) of these studies demonstrated sustained functional brain changes in the trained frequency ranges, which were often accompanied by transient reductions in self‐reported tinnitus distress and loudness (Neff and Meyer 2024). A possible reason for such inconsistencies could be that scalp EEG‐based neurofeedback training protocols rely on diffuse brain activity originating from multiple neural sources and therefore lack anatomical sensitivity (Elmer and Jäncke 2014). Nevertheless, methodological advances in EEG signal processing made it have possible to reconstruct current density in three‐dimensional brain space through source estimation algorithms that provide satisfactory spatial resolution (Pascual‐Marqui et al. 2002). Notably, such source estimation approaches have also found application in tinnitus research, and a growing number of studies have already utilized the spatial resolution of tomographic neurofeedback to specifically regulate brain activity in target regions such as the auditory cortex, the anterior insula, the anterior cingulate cortex, or even frontal and parietal areas (Congedo et al. 2004; Güntensperger et al. 2020; Hartmann et al. 2014; Vanneste et al. 2018). The main advantage of tomographic EEG neurofeedback is that it uses source localization algorithms to provide feedback from specific cortical regions rather than scalp electrodes alone, enabling more precise and targeted modulation of neural activity. In this context, Hartmann and colleagues (2014) used tomographic neurofeedback based on magnetoencephalography (MEG) to train alpha band up‐regulation in auditory areas over a period of approximately four weeks (two to three sessions per week) in a sample of tinnitus patients compared to a control group that received ten sessions of repeated transcranial magnetic stimulation (rTMS). Source‐level alpha power measured at rest before and after the interventions increased exclusively in the right auditory areas of the neurofeedback group. This training effect co‐occurred with increased functional connectivity profiles in the proximity of the auditory cortex and was associated with a reduction in tinnitus symptoms. Using EEG, Güntensperger et al. (2020) applied alpha up‐ and delta down‐regulation training over 15 once‐weekly sessions to two groups of individuals with tinnitus who received either tomographic‐ (four voxels located in the primary auditory cortex) or electrode‐based neurofeedback. In both groups, the resting‐state alpha/delta ratio increased as a function of training and remained stable during the follow‐up period, with no apparent differences between the two protocols. Furthermore, both interventions resulted in significant reductions in tinnitus loudness and distress, although changes in distress persisted and loudness returned to baseline during the follow‐up period. However, some studies have also failed to demonstrate a measurable effect of tomographic neurofeedback on brain activity or functional connectivity metrics (Congedo et al. 2004; Vanneste et al. 2018). Congedo and colleagues (2004) conducted six to twenty EEG‐based tomographic neurofeedback sessions over several weeks in a small sample of six participants with tinnitus to increase beta/alpha power ratio in the anterior cingulate cortex. Although beta power increased significantly after training, the authors found no effect of the intervention on the trained power ratio in the target region. Similarly, Vanneste et al. (2018) applied 15 sessions of tomographic EEG neurofeedback to modulate alpha (up‐regulation), beta and gamma (down‐regulation) power in the posterior cingulate cortex, and reduce tinnitus‐related distress, but the training protocol had no influence on these specific electrophysiological features. Nevertheless, tinnitus‐related distress decreased significantly after training, and this behavioral index was accompanied by changes in functional connectivity surrounding the trained brain region.

### 1.4 Authors’ Previous Neurofeedback Studies in Tinnitus and the Present Study

The present work represents an extension of two previous EEG neurofeedback studies from our group with the aim of increasing the alpha/delta power ratio at the scalp level (Güntensperger et al. 2020; Güntensperger et al. 2019) or in the auditory cortex (Güntensperger et al. 2020) by application of one weekly neurofeedback session. Unlike in the previous studies, the total number of NFB sessions per participant in the current study was reduced to 12 sessions. Although the two previous studies provided evidence of an influence of neurofeedback training on the alpha/delta power ratio, long‐lasting EEG effects after the intervention varied between 1 week (Güntensperger et al. 2019) and 3 months (Güntensperger et al. 2020). Furthermore, self‐reported tinnitus distress generally decreased after neurofeedback, but follow‐up stability varied in the range of 3 (Güntensperger et al. 2020) to 6 (Güntensperger et al. 2019) months, and tinnitus loudness consistently returned to baseline shortly after the intervention. Several explanations may account for these inconsistent findings. First, one possible reason for such variable outcomes could be that the neurofeedback training only targeted a small portion of the neural tinnitus network and hence mistargeted other relevant hubs in the tinnitus brain (De Ridder, Vanneste, et al. 2015; Güntensperger et al. 2017; Langguth et al. 2013; Meyer et al. 2017). Second and of greater importance, the most serious challenge for the diagnosis and intervention of chronic subjective tinnitus is its considerable heterogeneity (Cederroth et al. 2024). Tinnitus patients differ significantly in individual characteristics, for example, in stress, symptoms, coping mechanisms, and comorbidities such as sleep disorders, but also other factors such as years since the onset of tinnitus, education, or causal risk factors for hearing problems. All these factors, either individually or in various combinations, may shape the tinnitus brain in auditory and/or extra‐auditory areas (insula, anterior and posterior cingulate, hippocampal region) that are part of the tinnitus network, resulting in a variety of behavioral and neural states that would require individual protocols. There is currently no reliable specific diagnosis based on different types of tinnitus, even though the first steps in this direction have been made (Riha et al. 2022; Riha, Güntensperger, Kleinjung, and Meyer 2020; Riha et al. 2021). Therefore, it is not unlikely that a particular EEG neurofeedback protocol used in a clinical trial may help some participants but fail to modulate the neural patterns associated with tinnitus symptoms in other individuals. Finally, it is still unclear whether tinnitus sufferers would benefit from more training intensity or a longer intervention period and whether this has an influence on long‐term outcomes.

With the aim of modulating multiple auditory and extra‐auditory brain regions of the tinnitus network, we designed a longitudinal EEG study in which we aimed to leverage the potential of multifocal tomographic neurofeedback in two groups of older individuals with tinnitus (*N* = 30) attending one (*n* = 14, low‐intensity group, LI) or two (*n* = 16, high‐intensity group, HI) training sessions per week as part of a crossover experimental design. One group completed two training sessions per week in the first month and only one in the following month (HI), while the other group received the opposite treatment intensity (LI) with the same number of total neurofeedback sessions. Importantly, during each neurofeedback session, participants learned to sequentially modulate three, rather than just one, brain regions that are part of the perception, valence‐arousal, and salience networks. Specifically, the training protocol consisted of increasing the alpha/delta power ratio in the bilateral auditory cortex as well as the theta/beta ratio in the dorsal anterior cingulate cortex and the anterior insula. The effectiveness of tomographic neurofeedback and training intensity on resting‐state EEG parameters, as well as their influence on subjective tinnitus (distress, loudness, occurrence, and annoyance), psychological and health‐related dimensions (quality of life and health parameters), was assessed at multiple time points based on data collected before (T1) and after 1 month of training (T2), after 2 months of training (T3), and 3 (follow‐up 1, T4) and 6 (follow‐up 2, T5) months after the last training session. Based on a previous tomographic neurofeedback study from our group (Güntensperger et al. 2020), in which participants completed a comparable number of total training units over a 2‐month period, we expected a significant decrease in tinnitus loudness and distress from T1 to T3 with a normalization of loudness, but not necessarily distress, to the initial value in the two follow‐up measurements (T4 and T5). In addition, we predicted a general influence of neurofeedback training on the resting‐state power ratios extracted from the three target regions, as well as an effect of training intensity on EEG metrics and subjective psychometric variables.

## 2 Materials And Methods

### 2.1 Participants

The minimum number of participants needed for this clinical trial was estimated using the G*Power software (Faul, Erdfelder, Buchner, and Lang 2009) in conjunction with repeated measures analyses of variance (rANOVA). This procedure determined that the sample size required to detect a medium effect with a power of 0.80 should consist of at least 34 total participants. Based on this estimate and considering possible dropouts from the study, 39 individuals with chronic tinnitus aged 50–85 years and with symptoms that lasted longer than half a year were recruited at the Department of Otorhinolaryngology of the University Hospital of Zurich and via a database at the University of Zurich. The participants were proficient in German, did not wear cochlear implants, and had no history of psychiatric or neurological disorders. In addition, none of the participants showed signs of drug or alcohol dependence or took sedatives, neuroleptics, or antiepileptic drugs. From this sample of 39 participants, 9 had to be excluded due to dropouts during the COVID‐19 pandemic, resulting in a total sample size of 30 participants. Specifically, 1 participant did not complete the T3 measurement, 2 could not be measured at T4, 4 missed the measurement at T5, and 2 did not complete T4 and T5. According to an allocation scheme established before the start of the study, participants were matched by gender, age, and pure‐tone HL (pure‐tone average across both ears, PTA), and 14 participants were randomly assigned to the LI training group (mean age = 63.79 years, SD = 7.85; 6 women; PTA = 29.86, SD = 7.71), while 16 belonged to the HI group (mean age = 62.94 years, SD = 7.74; 6 women; PTA = 29.74, SD = 7.26). A random allocation sequence was generated and stored in anonymous form on a computer, and only DT and MM had access. The subjects were paid for participation, and the study was approved by the local ethics committee (Cantonal Ethics Committee, Project KEK‐ZH‐Nr. 2018‐01035) and registered online as a clinical trial at ClinicalTrials.gov (NCT03895047) and kofam.ch (SNCTP000003659). All methods were performed in accordance with the relevant guidelines and regulations. The consensus on the reporting and experimental design of the clinical and cognitive‐behavioral neurofeedback studies best‐practices checklist is provided in the Supplementary Materials.

### 2.2 Experimental Procedure

The study was conducted in the laboratory of the University of Zurich. At the first visit, participants were fully informed about the purpose of the study screened for inclusion and exclusion criteria, and signed their informed consent in the presence of a qualified healthcare professional. Subsequently, at the second visit, 1 to 2 weeks before the first neurofeedback training session, participants underwent a brief cognitive screening using the Montreal Cognitive Assessment (MoCA) to minimize the risk of including participants with substantial cognitive deficits as well as an audiometric measurement in which pure‐tone hearing thresholds at 0.25, 0.5, 1, 2, 4, 6, and 8 kHz were determined. Furthermore, each participant underwent a baseline resting‐state EEG measurement (T1) to define the frequency range of the individual alpha band (see section “neurofeedback training”) and completed demographic questionnaires. After collecting baseline EEG and psychometric data, participants began the clinical trial, which consisted of four additional measurement points, namely T2, T3, T4 (follow‐up 1), and T5 (follow‐up 2). The T2 measurement was performed after one month of neurofeedback training to assess the short‐term influence of neurofeedback and training intensity on resting‐state EEG data and psychometric dimensions. Following the study phase, participants were enrolled in a post‐study measurement (T3) one week after completion of the two‐month study phase, which included a resting‐state EEG and the collection of a reduced set of tinnitus‐related and other psychometric variables. Finally, the same procedure was repeated approximately three (T4) and six (T5) months after the end of neurofeedback training to assess the stability of the results over time.

The questionnaire set administered at baseline (T1) consisted of a variety of subjective instruments applied based on the Tinnitus Research Initiative (TRI) guidelines. An adapted version of the Tinnitus Sample Case History Questionnaire (TSCHQ) (Langguth et al. 2007) was used to collect demographic data, tinnitus characteristics (e.g., origin, location, loudness, and type), previous treatment attempts, and other tinnitus‐related questions. Two additional questionnaires were used to assess tinnitus distress (range 0–100), namely the German version of the Tinnitus Handicap Inventory (THI) (Kleinjung et al. 2007) and of the Tinnitus Functional Index (TFI) (Brüggemann et al., 2017). Participants also completed the German versions of Beck's Depression Inventory (BDI), Beck's Anxiety Inventory (BAI) (Beck et al. 1988), the short form of the WHO Quality of Life scale (WHOQOL‐BREF) (Angermeyer, 2000), and the Short Form Health Questionnaire (SF‐36) (Bullinger et al. 1995). Completing the questionnaires took approximately 45 min and was carried out on the computer while preparing the EEG. At screenings at T2, T3, T4, and T5, BAI and BDI were not administered, and the WHOQOL‐BREF was used only at T1 and T5. Consistent with previous work from our group (Güntensperger et al. 2020; Güntensperger et al. 2019), the main behavioral outcome measures of this study were loudness, occurrence, and annoyance of tinnitus (range 0–100) measured with the TSCHQ questionnaire, as well as tinnitus distress based on total scores from the THI and TFI surveys. We were also interested in examining the influence of neurofeedback on quality of life and health parameters using the WHO Quality of Life scale (WHOQOL‐BREF) and the Short Form Health Questionnaire (SF‐36). Regarding the latter, we focused the analyses exclusively on the “general health” and “physical functioning” sub‐scores.

### 2.3 Neurofeedback Training

**FIGURE 1:**
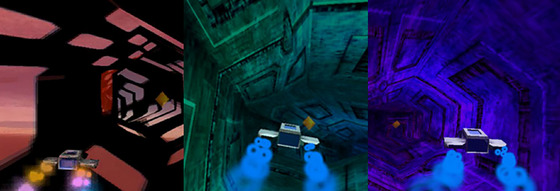
Schematic representation of the three spaceship visualization scenarios used in the neurofeedback experiment.

In the first month, the LI group received one neurofeedback training per week, while the HI group had two sessions. In the second month, the number of weekly training units alternated between the two groups so that each participant completed a total of 12 training sessions over the 2‐month period. The purpose of the neurofeedback training was to increase predetermined power ratios in three bilateral brain regions, namely the alpha/delta in the auditory cortex and the theta/beta ratio in the dorsal anterior cingulate cortex and the anterior insula. Visual feedback was implemented in the computer simulation Inner Tube (Somatic Vision, Encinitas, CA, USA), where participants observed a spaceship whose speed, navigation accuracy, and visibility changed according to their brain activity (Figure 1). An increase in alpha or theta power was rewarded, whereas increased delta or beta power was inhibited. The rewarded alpha frequency band was set in the range of ±2 Hz around the individual alpha peak determined for each participant at baseline (T1), whereas the rewarded theta frequency was in the range of 4–7 Hz. Furthermore, the inhibited delta and beta bands were defined in the ranges of 3–4 and 13–21 Hz, respectively. Since the tomographic neurofeedback technique used in this study was the same as that of a previous work of our group (Güntensperger et al. 2020), in the next paragraphs we reiterated some passages of the description of the procedure used in this previous study.

The neurofeedback data were collected using a NeuroAmp amplifier (BEE Medic GmbH, Singen, Germany) and 31 silver/silver chloride electrodes placed at the surface of the scalp according to the 10/20 system. The EEG signal was referenced to two electrodes attached to the earlobes, and the ground electrode was at the sensor position AFz. The sampling rate was 500  Hz, and the impedance was kept below 20  kΩ. The EEG was processed in real time using the software Cygnet 2.0.3.34 (BEE Medic GmbH, Singen, Germany), which automatically filtered the signal and excluded motion artifacts and system voltage fluctuations from the feedback (45–55 Hz).

Beyond the 45–55 Hz notch filter for line‐noise removal, the BEE Lab software (Cygnet 2.0.3.34) employed an automatic artifact detection algorithm for real‐time signal processing during neurofeedback sessions. When fast transients (e.g., due to movement or electrode artifacts) were detected, the system excluded the subsequent EEG segment from signal processing until the measured values returned to baseline after the transient. This approach ensured that contaminated data did not influence the real‐time feedback provided to participants. We deliberately chose not to implement additional offline correction methods such as independent component analysis (ICA) for eye blink artifact removal, despite having 31 EEG channels available for such procedures. This decision was based on several considerations: First, there was no prior evidence regarding the impact of ICA‐based artifact correction on source‐localized neurofeedback performance. Second, preliminary analyses indicated practically no influence of eye artifacts on the deep cortical sources being trained (auditory cortex, anterior insula, and dorsal anterior cingulate cortex). Third, movement artifacts were expected to be minimal due to the use of a full electrode cap in a controlled laboratory setting. Muscle artifacts were managed through direct observation of the EMG signal, which was continuously displayed to the neurofeedback therapist. When elevated muscle activity was detected, the therapist provided postural guidance to participants until muscle activity decreased. Importantly, the frequency bands selected for reward (alpha: individual peak ±2 Hz; theta: 4–7 Hz) and inhibit (delta: 3–4 Hz; beta: 13–21 Hz) were sufficiently distant from the typical EMG spectrum (>30 Hz), minimizing potential contamination from muscle activity. The hardware architecture contributed significantly to signal quality. The NeuroAmp amplifier provided a 1 MHz gross sampling rate with 24‐bit resolution, resulting in excellent aliasing performance. With an analog bandwidth of 160 Hz and a data processing rate of 500 samples/s, the system effectively eliminated high‐frequency noise and artifacts from the signal chain. Built‐in filters removed all activity above the gamma range from the signal processing pipeline, ensuring that only clean signals within the frequency bands of interest were used for neurofeedback training.

All 31 electrodes were considered for the calculation of the inverse solution based on eLORETA algorithms and a high regularization transformation matrix (Pascual‐Marqui 2002). Using this method, we estimated the target signal in four voxels located (Talairach coordinates) in the bilateral primary auditory cortex (x = 55, y = −25, z = 10; x = 55, y = −30, z = 10; x = −55, y = −25, z = 10; x = −55, y = −30, z = 10), the dorsal anterior cingulate cortex (x = 5, y = 0, z = 40; x = 5, y = 0, z = 35; x = ‐5, y = 0, z = 40; x = ‐5, y = 0, z = 35), and the anterior insula (x = 40, y = −10, z = 10; x = 40, y = −15, z = 10; x = −40, y = −10, z = 10; x = −40, y = −15, z = 10). Only brain activity emanating from these target regions was considered for the neurofeedback sessions.

A neurofeedback therapist experienced in tomographic neurofeedback monitored visual feedback during the initial training sessions and adjusted inhibition and reward thresholds to maintain an approximate success rate in the 80–90% range. For subsequent sessions, the therapist logged the threshold changes to begin each new session with the most recently recorded thresholds from the previous session to better monitor neurofeedback progress over time. During each neurofeedback session, participants trained the three brain regions in a sequential order that changed from session to session based on a randomization scheme and was counterbalanced across individuals. Participants were not informed about the brain regions being trained, nor were they told in which order the three brain regions would be monitored. However, to facilitate the effectiveness of training as well as switching between the three regions of interest (ROIs) and the different frequency bands to be trained, three visually distinguishable designs were selected to provide feedback during each session (Somatic Vision, Encinitas, CA, USA). Before the first training began, each participant had the opportunity to choose three designs from a larger selection that was maintained over the study period. In each design, participants observed a flying object navigating through a tunnel at a specific speed, and navigation accuracy and visibility were directly influenced according to recorded brain activity. The only differences between the three designs were the tunnel color and the type of flying object (e.g., spaceship, airplane, butterfly). Importantly, increasing alpha or theta power was rewarded with ship acceleration, but increasing delta or beta power reduced autopilot accuracy and simulation visibility, regardless of the brain region trained. Each brain region was trained for seven minutes, with a total of 21 minutes of training per session.

### 2.4 Resting‐State EEG Data Acquisition

A BrainAmp DC amplifier system with a 64 active channel actiCap electrode cap (Brain Products, Munich, Germany) and a sampling rate of 1000 Hz was used to record the resting‐state EEG at T1, T2, T3, T4, and T5. The silver/silver chloride sensors were placed over the scalp using the 5/10 electrode position system (Oostenveld and Praamstra 2001). The electrode FCz was used as an online reference, and a ground electrode was positioned at AFz. The electrodes’ impedance was kept below 10 kΩ using electrically conductive gel, and recordings were made in direct current (DC) mode with a high‐frequency cutoff filter of 1000 Hz and a slope of 12 dB/octave. During recording, subjects were instructed by a pre‐recorded voice to open (EO) and close (EC) their eyes at regular intervals using the Presentation software (Neurobehavioral Systems Inc., 2010), and a fixation cross was presented during the eyes‐open condition. Resting‐state EEG was recorded twice over a period of 8 min. While no additional instructions were given in the first 8 minutes of recording (EEG with no task), in the second measurement the patients were asked not to consciously suppress their tinnitus (EEG with task). This was done to control confounding suppression effects that in tinnitus sufferers (Sedley and Cunningham 2013). Importantly, in accordance with the guidelines of the Tinnitus Research Initiative (TRI) and the recommendation of Working Group 3 of the European Tinnitus Research Network (TINNET, http://www.tinnet.tinnitusresearch.net/), we only assessed the eyes‐open resting‐state condition with explicit instructions not to suppress the tinnitus. The other resting‐state periods were recorded to answer research questions unrelated to this work and were therefore not evaluated.

### 2.5 EEG Data Processing

The resting‐state EEG data were preprocessed using the Brain Vision Analyzer software package (version 2.0.1, Brain Products GmbH, D‐82205 Gilching). initially, the raw EEG data were re‐referenced to an average reference and filtered with a band‐pass filter (48 dB/octave, Butterworth zero‐phase filter) of 0.1–30 Hz, including a notch filter of 50 Hz. Noisy channels were interpolated, and eye movements were corrected by applying independent component analysis (ICA) implemented in the Brain Vision Analyzer toolbox. Remaining muscle artefacts were removed from −200 ms before to 200 ms after events using an automatic raw data inspection if a voltage gradient criterion of 50 µV/ms or an amplitude criterion of ±100 µV was exceeded. Afterwards, the EEG data of the eyes‐open condition with instruction not to suppress tinnitus were segmented into 2 s epochs with 1 s overlap and exported to the eLORETA toolbox (version 20140711, http://www.uzh.ch/keyinst/loreta.htm) for source‐based analyses (Pascual‐Marqui 2002).

The eLORETA software estimates the three‐dimensional distribution of electrical activity in the brain as a current density value (µA/mm^2^) and provides a solution to the inverse problem by assuming that the smoothest of all possible activity distributions is the most plausible one for explaining the data (Pascual‐Marqui 2002). The characteristic feature of this inverse solution approach is the low spatial resolution, which conserves the location of maximal activity but with a certain degree of dispersion (Mulert et al. 2004). Technical details on eLORETA can be found elsewhere (Pascual‐Marqui et al. 2002; Pascual‐Marqui et al. 2011). In the eLORETA toolbox, the segmented data were transformed into the frequency domain using fast Fourier transforms (FFT) coupled with a Hanning window. Subsequently, for each segment, the spectral density values for each voxel were calculated according to the predefined frequency bands of interest using the “exact LORETA” transformation matrix and a frequency resolution of 0.5  Hz. The alpha frequency band was individually adjusted in the range of ±2 Hz around the individual peak determined for each participant at T1. In the next step, the spectral density values for each participant were averaged across all segments, subject‐wise normalized, and in the eLORETA toolbox. Finally, for each ROI, mean spectral density values corresponding to the predefined frequency bands were extracted to compute the alpha/delta power ratio in the bilateral auditory cortex as well as the theta/beta ratio in the dorsal anterior cingulate cortex and the anterior insula.

### 2.6 Statistical Analyses

The statistical analyses were grouped into three main clusters and included (1) group comparisons at baseline (T1), as well as (2) analysis of EEG metrics and (3) subjective questionnaire data as a function of neurofeedback across all time points (T1–T5). All statistical analyses were conducted using IBM SPSS Statistics 26 software package (SPSS, an IBM company, Armonk, New York, USA). To ensure that the two groups were properly matched, initially (1) we calculated a series of t‐tests (two‐tailed, Bonferroni‐corrected) to compare subjective tinnitus characteristics as well as biographical, audiometric, and health‐related data at baseline between the two groups. Assessment of subjective tinnitus characteristics at T1 focused on tinnitus loudness, occurrence, annoyance, duration (TSCHQ), and distress (THI and TFI). Additionally, we limited the analyses for the other influencing variables to age, Beck's anxiety (BAI) and depression (BDI) scores, general health (SF36), physical functioning (SF36), and the Quality of Life scale (WHOQOL‐BREF). In the next analysis step (2), the EEG resting‐state ratio values were evaluated using 2 × 5 rANOVAs with the factors group (LI and HI) and measurement time point (T1–T5), separately for each ROI. Significant main and interaction effects were further examined by post‐hoc rANOVAs or *t*‐tests (two‐tailed) and corrected for multiple comparisons using the Bonferroni procedure. Finally (3), each of the key behavioral outcome variables, namely tinnitus loudness, occurrence, annoyance and distress, general health and physical functioning, was evaluated by 2 × 5 rANOVAs (two groups and 5 time points). However, because quality of life was only measured at T1 and T5, this dimension was assessed using a 2 × 2 rANOVA (2 groups and 2 time points). In the case of missing data, participants were not included in the analyses for the relevant dimensions, ensuring that results were based only on complete data.

## 3 Results

### 3.1 Group Comparisons of Subjective Tinnitus Characteristics at Baseline (T1)

Before subjecting the tinnitus‐related data to statistical analysis, we provide a descriptive overview of the tinnitus characteristics of the two samples. Of the 14 participants in the LI group, 9 described their tinnitus perception as tonal, 2 as noise‐like, and 3 reported a mixed type (e.g., rushing, humming, whizzing, whistling, and hissing). 9 participants in the HI group (*n* = 16) perceived tinnitus as tonal, 4 as noise‐like, and 3 mentioned a mixed type of tinnitus. According to the TSCHQ questionnaire, 1 participant in the LI group reported tinnitus in both ears with worse sensation on the left side, 6 in both ears with worse sensation on the right side, 4 in both ears equally, 2 inside the head, and 1 elsewhere. In contrast, in the HI group, 1 participant had tinnitus in the right ear, 1 in the left ear, 5 in both ears with worse sensation on the left, 6 in both ears equally, and 3 inside the head. The reported heterogeneity in tinnitus sensation (sound, lateralization) is typical for samples of tinnitus patients and is usually not controlled in clinical studies. The subjective loudness, occurrence and annoyance in the last month (range 0–100) and duration of tinnitus were assessed using the TSCHQ questionnaire, while tinnitus distress was estimated by means of the TFI and THI questionnaires. However, as expected and due to the matching procedure used, independent samples *t*‐tests (Bonferroni‐corrected *p*‐value for 6 comparisons = 0.0083) at baseline did not reveal a significant difference between the two groups for any of the tested variables (Table 1). In particular, the two groups were characterized by comparable tinnitus loudness. The two groups also showed similar mean occurrences of tinnitus and tinnitus annoyance, although the latter variable was apparently slightly higher in the HI cohort. The subjective data also demonstrated a clear self‐reported improvement of chronic tinnitus in both groups, with a mean tinnitus duration of 263.23 months in the LI group and 171.43 months in the HI group. Information on tinnitus duration was missing for one participant in the LI group and for two participants in the HI group. At baseline both groups also showed moderate (TFI) to mild (THI) tinnitus distress (HI group: THI mean = 27.12, SD = 17.29; TFI mean = 29.92, SD = 17.63; LI group: THI mean = 20.71, SD = 13.91; TFI mean = 24.37, SD = 16.13) with no significant differences between the two cohorts.

**TABLE 1:** Group comparisons of relevant variables at baseline (T1).

Baseline variable	Mean LI	Mean HI	SD LI	SD HI	df	*t*‐values	*p*‐value
Age	63.79	62.94	7.85	7.74	28	0.297	0.768
PTA	29.86	29.74	7.71	7.26	28	0.045	0.964
BAI	3.57	5.63	3	5.47	28	−1.247	0.223
BDI	4.64	6.06	4.23	6.96	28	−0.662	0.513
General health	66.79	67.81	17.38	18.79	28	−0.155	0.878
Physical functioning	94.64	92.81	6.64	9.65	28	0.596	0.556
Quality of life	80.35	72.65	16.04	17.21	28	1.261	0.218
Tinnitus loudness	50.21	50.56	18.64	23.74	28	−0.044	0.965
Tinnitus occurrence	44.64	43.38	28.04	27.78	28	0.124	0.902
Tinnitus annoyance	17.79	24.75	22.36	23.63	28	−0.826	0.416
Tinnitus duration	263.23	171.43	221.3	156.06	25	1.253	0.222
Tinnitus distress TFI	24.37	29.92	16.13	17.63	28	−0.895	0.378
Tinnitus distress THI	20.71	27.13	13.91	17.29	28	−1.108	0.277

**Abbreviations**: df = degrees of freedom, HI = high‐intensity group, LI = low‐intensity group, SD = standard deviation.

### 3.2 Group Comparisons of Other Relevant Variables at Baseline (T1)

To further ensure group homogeneity at baseline on variables other than tinnitus, we compared a specific set of demographic, audiometric, and health‐related dimensions between the two groups using 7 independent samples *t*‐tests (Bonferroni‐corrected *p*‐value for 7 tests = .0071), namely age, PTA, Beck's anxiety (BAI) and depression (BDI) scores, general health (SF36), physical functioning (SF36) and the Quality of Life (WHOQOL‐BREF). However, none of the comparisons reached significance (Table 1), indicating group comparability at the beginning of the study.

### 3.3 Resting‐State EEG Data as a Function of Time Points (T1–T5)

The main aim of this study was to assess putative changes in spectral power ratios in three specific brain regions. Because the training specifically aimed at increasing the alpha/delta power ratio in the bilateral auditory cortex as well as the theta/beta ratio in the dorsal anterior cingulate cortex and the anterior insula, we focused the main analyses on these parameters. Accordingly, these three source‐based power ratios were compared between the two groups using separate 2 × 5 rANOVAs with the factors group and measurement time point (T1–T5). However, contrary to our hypothesis, we did not find any main effects of time point (auditory cortex: *F*_(1.288, 36.071)_ = 0.776, *p* = 0.415, partial eta^2^ = 0.027; dorsal anterior cingulate cortex: *F*_(2.990, 83.730)_ = 0.628, *p* = 0.599, partial eta^2^ = 0.022; insula: *F*_(2.893, 81.008)_ = 0.710, *p* = 0.544, partial eta^2^ = 0.025) or time point x group interaction effects (auditory cortex: *F*_(1.288, 36.071)_ = 0.734, *p* = 0.430, partial eta^2^ = 0.026; dorsal anterior cingulate cortex: *F*_(2.990, 83.730)_ = 0.023, *p* = 0.995, partial eta^2^ = 0.001; insula: *F*_(2.893, 81.008)_ = 0.596, *p* = 0.613, partial eta^2^ = 0.021). The main effects of group also did not reach significance (auditory cortex: *F*_(1, 28)_ = 3.288, *p* = 0.081, partial eta^2^ = 0.105; dorsal anterior cingulate cortex: *F*_(1, 28)_ = 0.249, *p* = 0.622, partial eta^2^ = 0.009; insula: *F*_(1, 28)_ = 2.908, *p* = 0.099, partial eta^2^ = 0.094).

**FIGURE 2:**
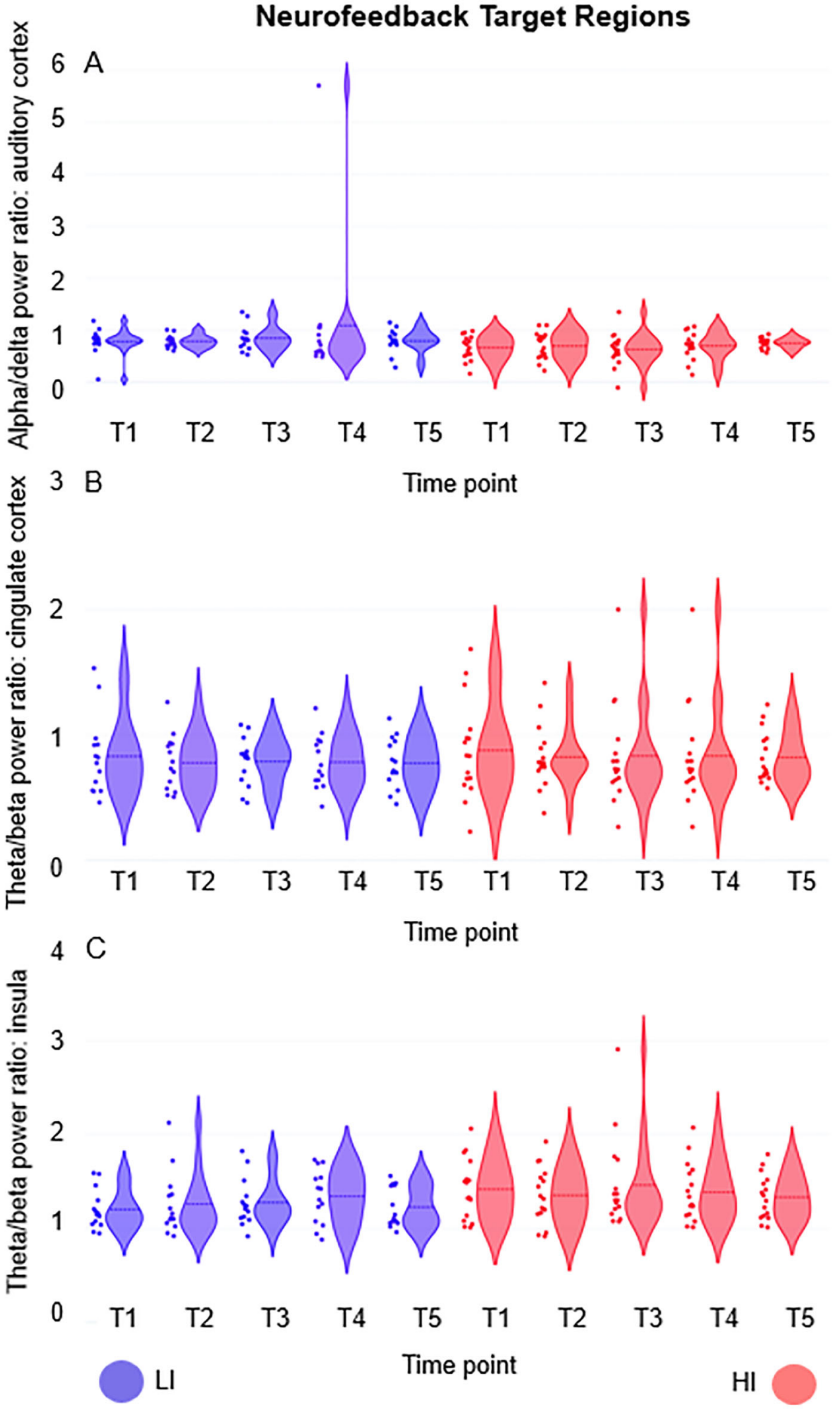
Single‐subject data and violin plots with density distribution and mean. EEG power ratios in the three neurofeedback target regions as a function of time points (T1–T5) and groups (blue = low‐intensity, LI; red = high‐intensity, HI). A = up‐regulation of alpha/delta power ratio in the auditory cortex, B = up‐regulation of theta/beta power ratio in the dorsal anterior cingulate cortex, C = up‐regulation of theta/beta power ratio in the anterior insula.

Based on these results, we performed additional explorative analyses to rule out that the ratio values obscure possible modulations in individual frequency bands that were not manifested in the quotient metrics. Accordingly, we calculated separate 2 × 5 rANOVAs for each of the three ROIs, with the values ​​of the two frequency bands initially included in the ratio calculations (Bonferroni‐corrected *p‐*value for two comparisons = 0.025). However, neither individual alpha (time point: *F*_(4, 112)_ = 0.216, *p* = 0.929, partial eta^2^ = 0.008; time point × group: *F*_(4, 112)_ = 1.307, *p* = 0.272, partial eta^2^ = 0.045; group: *F*_(1, 28)_ = 0.941, *p* = 0.340, partial eta^2^ = 0.033) and delta (time point: *F*_(4, 112)_ = 0.107, *p* = 0.980, partial eta^2^ = 0.004; time point × group: *F*_(4, 112)_ = 0.526, *p* = 0.717, partial eta^2^ = 0.018; group: *F*_(1, 28)_ = 0.775, *p* = 0.386, partial eta^2^ = 0.027) power in the auditory cortex nor theta (time point: *F*_(4, 112)_ = 1.943, *p* = 0.108, partial eta^2^ = 0.065; time point x group: *F*_(4, 112)_ = 0.607, *p* = 0.659, partial eta^2^ = 0.021; group: *F*_(1, 28)_ = 1.012, *p* = 0.323, partial eta^2^ = 0.035) and beta (time point: *F*_(4, 112)_ = 1.787, *p* = 0.136, partial eta^2^ = 0.060; time point × group: *F*_(4, 112)_ = 0.676, *p* = 0.610, partial eta^2^ = 0.024; group: *F*_(1, 28)_ = 0.714, *p* = 0.405, partial eta^2^ = 0.025) power in the dorsal anterior cingulate cortex or theta (time point: *F*_(4, 112)_ = 0.952, *p* = 0.437, partial eta^2^ = 0.033; time point × group: *F*_(4, 112)_ = 0.144, *p* = 0.965, partial eta^2^ = 0.005; group: *F*_(1, 28)_ = 0.180, *p* = 0.675, partial eta^2^ = 0.006) and beta (time point: *F*_(2.842, 79.585)_ = 1.597, *p* = 0.199, partial eta^2^ = 0.054; time point × group: *F*_(2.842, 79.585)_ = 0.534, *p* = 0.651, partial eta^2^ = 0.019; group: *F*_(1, 28)_ = 1.578, *p* = 0.219, partial eta^2^ = 0.053) power in the anterior insula reached significance. In summary, the statistical analyses showed no evidence of an influence of neurofeedback on the trained brain regions and frequency bands. The EEG results are summarized in Table 2 and Figure 2.

**TABLE 2:** Group comparisons of the resting‐state data as a function of time points (T1–T5).

EEG parameters	Brain region	rANOVA factor	df	*F*‐values	*p*‐value
Alpha/delta	Auditory cortex	Time point	1.288, 36.071	0.776	0.415
		Group	1, 28	3.288	0.081
		Group × time point	1.288, 36.071	0.734	0.43
Theta/beta	Anterior cingulate cortex	Time point	2.990, 83.730	0.628	0.599
		Group	1, 28	0.249	0.622
		Group × time point	2.990, 83.730	0.023	0.995
Theta/beta	Insula	Time point	2.893, 81.008	0.71	0.544
		Group	1, 28	2.908	0.099
		Group × time point	2.893, 81.008	0.596	0.613
Alpha	Auditory cortex	Time point	4, 112	0.216	0.929
		Group	1, 28	0.941	0.34
		Group × time point	4, 112	1.307	0.272
Delta	Auditory cortex	Time point	4, 112	0.107	0.98
		Group	1, 28	0.775	0.386
		Group × time point	4, 112	0.526	0.717
Theta	Anterior cingulate cortex	Time point	4, 112	1.943	0.108
		Group	1, 28	1.012	0.323
		Group × time point	4, 112	0.607	0.659
Beta	Anterior cingulate cortex	Time point	4, 112	1.787	0.136
		Group	1, 28	0.714	0.405
		Group × time point	4, 112	0.676	0.61
Theta	Insula	Time point	4, 112	0.952	0.437
		Group	1, 28	0.18	0.675
		Group × time point	4, 112	0.144	0.965
Beta	Insula	Time point	2.842, 79.585	1.597	0.199
		Group	1, 28	1.578	0.219
		Group × time point	2.842, 79.585	0.534	0.651

**Abbreviation**: df = degrees of freedom.

### 3.4 Subjective Tinnitus and Health‐related Data as a Function of Time Points (T1–T5)

Consistent with previous work from our group, the neurofeedback intervention was additionally assessed using separate rANOVAs for each of the behavioral outcome variables, namely tinnitus loudness, occurrence, annoyance, and distress, as well as general health, physical functioning, and quality of life (Güntensperger et al. 2020; Güntensperger et al. 2019). Due to the COVID‐19 pandemic, some participants' data are missing, resulting in reduced sample sizes for each variable of interest, which will be reported separately for each analysis.

The 2 × 5 rANOVA computed on the subjective tinnitus loudness data (LI group n = 9; HI group n = 9) did not yield significant results (time point: *F*_(2.597, 41.551)_ = 2.426, *p* = 0.087, partial eta^2^ = 0.132; time point × group: *F*_(2.597, 41.551)_ = 0.244, *p* = 0.838, partial eta^2^ = 0.015; group: *F*_(1, 16)_ = 0.078, *p* = 0.784, partial eta^2^ = 0.005), and the same was true for the rANOVA calculated with the tinnitus occurrence (LI group *n* = 10; HI group *n* = 12; time point: *F*_(4, 80)_ = 2.227, *p* = 0.073, partial eta^2^ = 0.100; time point × group: *F*_(4, 80)_ = 0.382, *p* = 0.821, partial eta^2^ = 0.019; group: *F*_(1, 20)_ = 0.458, *p* = 0.507, partial eta^2^ = 0.022) and tinnitus annoyance metrics (LI group *n* = 9; HI group *n* = 12; time point: *F*_(2.875, 54.617)_ = 0.728, *p* = 0.534, partial eta^2^ = 0.037; time point × group: *F*_(2.875, 54.617)_ = 0.513, *p* = 0.667, partial eta^2^ = 0.026; group: *F*_(1, 19)_ = 0.007, *p* = 0.934, partial eta^2^ = 0.000). In contrast, the analysis of the THI (LI group *n* = 13; HI group *n* = 12) revealed a main effect of time point (*F*_(4, 92)_ = 4.951, *p* = 0.001, partial eta^2^ = 0.177), while the time point × group interaction (*F*_(4, 92)_ = 0.536, *p* = 0.710, partial eta^2^ = 0.023) and the main effect of group (*F*_(1, 23)_ = 2.674, *p* = 0.116, partial eta^2^ = 0.104) did not reach significance. Additional post‐hoc t‐tests for dependent samples between the different time points (Bonferroni‐corrected *p* value for 10 tests = .005) showed that the main effect of time point was due to lower THI values at T3 compared to T1 (*t*_(29)_ = 3.855, *p* < 0.001) and T2 (*t*_(26)_ = 3.995, *p* < 0.001), and this result showed a tendency to remain at the same low level until T4 (T2 vs. T4: *t*_(24)_ = 2.930, *p* = 0.007). Similarly, analysis of TFI scores (LI group *n* = 13; HI group *n* = 12) also revealed a main effect of time point (*F*_(4, 92)_ = 2.575, *p* = 0.043, partial eta^2^ = 0.101), while the time point x group interaction (*F*_(4, 92)_ = 0.287, *p* = 0.886, partial eta^2^ = 0.012) and the main effect of group (*F*_(1, 23)_ = 1.963, *p* = 0.175, partial eta^2^ = 0.079) did not reach significance. Post‐hoc *t*‐tests for dependent samples between the different time points did not survive the Bonferroni correction (Bonferroni‐corrected *p*‐value for 10 tests = .005), but the main effect of time point was related to lower TFI values at T3 (*t*_(26)_ = 2.665, *p* = 0.013) and T4 (*t*_(24)_ = 2.687, *p* = 0.013) compared to T2.

**FIGURE 3:**
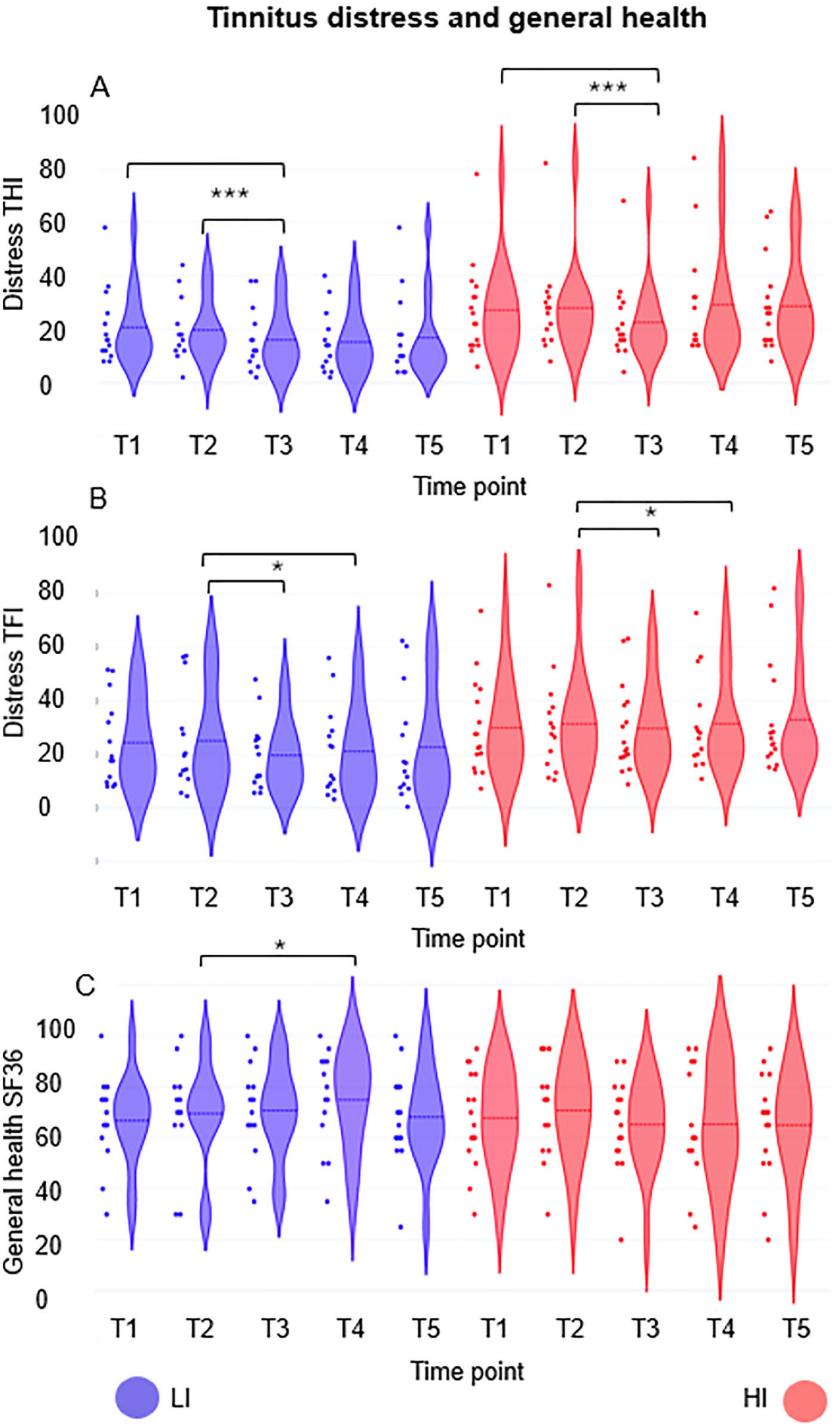
Single‐subject data and violin plots with density distribution and mean. Tinnitus distress and general health as a function of time points (T1–T5) and groups (blue = low‐intensity (LI), red = high‐intensity (HI). A = tinnitus distress (THI), B = tinnitus distress (TFI), and C = general health (SF36). ***p < 0.001 and *p < 0.05. Lower THI and TFI scores indicate amelioration of tinnitus symptoms, whereas an increase in SF‐36 scores reflects improvement in health parameters.

The 2 × 5 rANOVA calculated to evaluate general health scores (LI group *n* = 13; HI group *n* = 12) showed a significant group x time point interaction effect (*F*_(4, 92)_ = 2.534, *p* = 0.045, partial eta^2^ = 0.099), whereas the main effects of time point (*F*_(4, 92)_ = 0.936, *p* = 0.447, partial eta^2^ = 0.039) and group (*F*_(1, 23)_ = 0.004, *p* = 0.952, partial eta^2^ = 0.000) did not reach significance. Although dependent sample *t*‐tests calculated separately for the two groups across the 5 time points did not survive the Bonferroni correction (Bonferroni‐corrected *p*‐value for 10 tests = 0.005), the time point x group interaction effect was driven by the LI group, with higher health scores at T4 compared to T2 (*t*_(12)_ = −2.914, *p* = 0.013, all other *p*‐values > 0.08; HI group all *p*‐values > 0.144). Furthermore, the 2×5 rANOVA used to assess physical functioning (LI group *n* = 13; HI group *n* = 12) showed neither significant main effects of time point (*F*_(2.178, 50.086)_  = 2.031, *p* = 0.138, partial eta^2^ = 0.081) or group (*F*_(1, 23)_ = 0.122, *p* = 0.730, partial eta^2^ = 0.005) nor a significant interaction between time point and group (*F*_(2.178, 50.086)_ = 0.205, *p* = 0.833, partial eta^2^ = 0.009). Finally, also the assessment of quality of life (LI group *n* = 14; HI group *n* = 16) using a 2 × 2 rANOVA (2 groups × T1 and T5) did not produce significant results (time point: *F*_(1, 28)_ = 0.325, *p* = 0.573, partial eta^2^ = 0.011; time point × group: *F*_(1, 28)_ = 0.098, *p* = 0.757, partial eta^2^ = 0.003; group: *F*_(1, 28)_ = 1.377, *p* = 0.250, partial eta^2^ = 0.047). All results are summarized in Table 3 and Figure 3.

**TABLE 3:** Group comparisons of subjective tinnitus and health‐related variables as a function of time points (T1–T5).

Baseline variable	rANOVA factor	df	*F*‐values	*p*‐value
Tinnitus loudness	Time point	2.597, 41.551	2.426	0.087
	Group	1, 16	0.078	0.784
	Time point × Group	2.597, 41.551	0.244	0.838
Tinnitus occurrence	Time point	4, 80	2.227	0.073
	Group	1, 20	0.458	0.507
	Time point × Group	4, 80	0.382	0.821
Tinnitus annoyance	Time point	2.875, 54.617	0.728	0.534
	Group	1, 19	0.007	0.934
	Time point × Group	2.875, 54.617	0.513	0.667
Tinnitus distress THI	Time point	4, 92	4.951	**0.001**
	Group	1, 23	2.674	0.116
	Time point × Group	4, 92	0.536	0.71
Tinnitus distress TFI	Time point	4, 92	2.575	**0.043**
	Group	1, 23	1.963	0.175
	Time point × Group	4, 92	0.287	0.886
General health	Time point	4, 92	0.936	0.447
	Group	1, 23	0.004	0.952
	Time point × Group	4, 92	2.534	**0.045**
Physical functioning	Time point	2.178, 50.086	2.031	0.138
	Group	1, 23	0.122	0.73
	Time point × Group	2.178, 50.086	0.205	0.833
Quality of life	Time point	1, 28	0.325	0.573
	Group	1, 28	1.377	0.25
	Time point × Group	1, 28	0.098	0.757

**Abbreviation**: df = degrees of freedom.

## 4 Discussion

### 4.1 General Discussion

Previous studies based on sensor‐ (Crocetti et al. 2011; Dohrmann et al. 2007; Guillard et al. 2021; Güntensperger et al. 2019) or source‐level brain activity (Congedo et al. 2004; Hartmann et al. 2014; Neff and Meyer 2024; Vanneste et al. 2018) have provided inconsistent results on the effectiveness of EEG neurofeedback on neural and subjective psychometric tinnitus outcome variables. In the present clinical trial, we introduced a new approach and investigated the potential of multifocal tomographic neurofeedback to modulate brain activity in three bilateral cortical regions and several frequency bands, rather than focusing on individual sources. Furthermore, we examined the influence of HI and LI neurofeedback on neural parameters as well as psychological and health‐related variables. Contrary to our expectations, we were unable to detect short‐ or long‐term functional changes in neural activity collected from the trained brain regions over time, nor by evaluating EEG power ratios or power spectra in the individual frequency bands of interest. These null results notwithstanding, we noted significant reductions in tinnitus distress in both the HI and LI groups (TFI and THI questionnaires) after the training period, which tended to last until the first follow‐up measurement three months after the last training session. The implications of these results are discussed in more detail in the next sections.

### 4.2 Resting‐State EEG Data Over Time as a Function of Neurofeedback (T1–T5)

Based on recent data indicating an involvement of the auditory cortex, the insula, and the cingulate cortex in the perception and maintenance of tinnitus (De Ridder, Congedo, et al. 2015; Langguth et al. 2013; Meyer et al. 2017), the aim of this study was to alleviate the tinnitus symptoms through modulation of brain activity in these three brain regions. The auditory cortex has repeatedly been a topic of tinnitus research, primarily due to the salience of auditory phantom sensations and its structural changes associated with the syndrome (Elmer et al. 2023; Meyer et al. 2016; Schecklmann et al. 2013). In addition, tinnitus sufferers are often characterized by a reorganization of auditory cortical maps (Okamoto et al. 2010), a shift in spontaneous firing rates (Eggermont 2015), and dysfunctional neuronal activity in the auditory cortex (Ghazaleh et al. 2017). The cingulate cortex is another crucial brain area of the tinnitus network, which is thought to be involved in sustained attention to auditory sensations (De Ridder, Joos, and Vanneste 2016; Vanneste et al. 2024; Vanneste et al. 2010) and plays an important role in the burdensome and distressing nature of tinnitus (Vanneste and De Ridder 2012). The relevance of these two specific brain structures for tinnitus can also be derived from previous studies that used tomographic neurofeedback to modulate auditory areas (Güntensperger et al. 2020; Hartmann et al. 2014) or the anterior (Congedo et al. 2004) or posterior (Vanneste et al. 2018) cingulate cortex. Regarding the anterior insula, it has been repeatedly shown that this region plays an important role in tinnitus‐related anxiety and distress (Chen et al. 2023; Chen et al. 2017; Leaver et al. 2012; Moazami‐Goudarzi, Michels, Weisz, and Jeanmonod 2010) and conscious pain perception (Nieuwenhuys 2012). Furthermore, the anterior insula is part of a neural circuit associated with salience detection (Cauda et al. 2012), and in conjunction with the anterior cingulate cortex, the anterior insula forms the core of a salience network that facilitates the detection of important environmental stimuli (Menon and Uddin 2010). Taken together, the anterior cingulate‐insular circuit contributes to the aversive evaluation of internally generated tinnitus sounds, which is accompanied by a loss of inhibitory attentional control (Meyer et al. 2016). Nevertheless, the anterior insula has not yet been a primary target of tomographic EEG neurofeedback studies.

In the present work, we used an innovative multifocal tomographic method and tested two neurofeedback protocols that differed in the amount of weekly training intensity. Nevertheless, our approach, as we were unable to provide evidence of neural changes in the target regions across different time points or as a function of training intensity. Such a result was not expected and poses challenges for future multifocal tomographic neurofeedback applications to treat tinnitus symptoms manifested in the tinnitus brain. Given the lack of effectiveness of the two tomographic protocols, we will provide possible reasons for the unpredictable results and discuss suggestions for experimental improvements. The first consideration concerns the multifocal tomographic neurofeedback approach itself, with which we aimed to modulate brain activity in three bilateral regions and frequency ranges. In this context, it is quite possible that the trained brain regions and frequency bands corresponded to the tinnitus‐related neuronal deviations in our sample but did not take into account the high physiological and anatomical variability associated with tinnitus (Cardon et al. 2022; Zhang et al. 2020). Accordingly, before applying multifocal tomographic neurofeedback, it would be useful to identify the brain regions of the involved tinnitus network for each participant separately using functional magnetic resonance imaging (fMRI) protocols and to select a specific group of tinnitus sufferers with similar neural profiles. In an optimal experimental setup, this approach can also be combined with EEG to target not only the brain regions to be trained but also the specific frequency bands.

A second consideration that we cannot exclude is that the up‐ and down‐regulation of frequency‐specific parameters in the target regions was mediated by functional connectivity with brain regions not examined in this study. Such a perspective would fit with at least one previous fMRI study showing that multiple brain regions are involved in regulating brain activity in a single target ROI (Kopel et al. 2017). More specifically, using a multivariate decoding model to assess neurofeedback training effects across the brain, Kopel and colleagues (2017) provided evidence that the coactivation patterns of at least 16 untrained brain regions best described neurofeedback training effects in the auditory cortex. Hence, we cannot rule out that the modulation of the auditory cortex, anterior insula, and anterior cingulate cortex during each training session was primarily mediated by other brain regions. This reasoning would also fit with a previous study by Vanneste and colleagues (2018), who used tomographic neurofeedback training of the posterior cingulate cortex and found no modulation of brain activity in the target region but instead revealed functional connectivity changes between remote brain areas as well as altered cross‐frequency coupling in the posterior cingulate cortex. Future neurofeedback studies using a multifocal approach should therefore focus not only on brain activity in the monitored brain regions but also on their dynamic interactions with other brain areas.

Another aspect that needs to be carefully considered is the duration of the neurofeedback training sessions. In fact, in our study, each of the three brain regions was trained for seven minutes per session, while other authors used longer training protocols. For example, Güntensperger et al. (2020) trained participants to monitor brain activity in the bilateral auditory cortex for a duration of 15 min per session, while Vanneste and colleagues (2018) conducted approximately 30‐min training sessions and Congedo et al. (2004) used 45 min interventions. Consequently, it is conceivable that the training protocols applied in each neurofeedback session were too short to result in lasting resting‐state changes. It is also important to note that although a neurofeedback therapist monitored visual feedback during the initial training sessions and adjusted inhibition and reward thresholds to maintain a high success rate, we did not collect resting‐state data after each individual neurofeedback session. This would have been particularly useful to assess baseline shifts after each training unit, thereby estimating the short‐term effects of the individual neurofeedback applications. Furthermore, it would have been interesting to include an additional control group with sham feedback to better assess the effect of the training compared to a group in which learning to modulate specific brain regions was not possible. However, for ethical reasons, such an undertaking was not possible.

As a further consideration, we did not observe any influence of training intensity on brain metrics. Although this aspect is clearly under‐researched, there is at least some evidence from an exploratory study conducted with small samples that suggests that different training parameters such as the number and duration of sessions and the interval between them do not necessarily determine the neural outcome variables (Esteves et al. 2021). In particular, Esteves and colleagues (2021) compared the effects of upper alpha band modulation between an “intensive” training group that completed four sessions of about 37 min on consecutive days and a “sparse” group that performed six sessions of 25 min each over a period of 3 weeks. However, neither group showed significant changes in the trained frequency band over the entire training period, raising the question of which specific training parameters might have the greatest influence. Since the influence of various training parameters on neurofeedback applications in connection with tinnitus is still largely unexplored, some open points must first be clarified before multifocal tomographic neurofeedback can be further recommended as an applicable tool in tinnitus therapy. Ultimately, it cannot be ruled out that the older age of the participants is responsible for the lack of positive effects. Age‐related brain atrophy does not spare either the temporal or cingulate cortex, and therefore it stands to reason that neuroplastic changes induced by neurofeedback do not occur in older individuals to the extent that would be expected in younger participants.

### 4.3 Subjective Tinnitus and Health‐related Outcomes: Comparison Before (T1) and After (T5) Neurofeedback Training

Consistent with previous studies, we found a significant decrease in tinnitus distress after neurofeedback interventions (Güntensperger et al. 2020; Güntensperger et al. 2019). In fact, THI and TFI values were generally lower at T3 and T4 than at T2, and THI metrics were reduced at T3 compared to T1. Since tinnitus distress is a complex phenomenon that can be associated with several negative psychological states such as anxiety, depression and irritability (Brüggemann et al. 2016; McKenna et al. 2014; Zirke et al. 2013), even a minimal improvement in this variable could contribute to a reduction in the perception of tinnitus and therefore have a positive effect on life satisfaction. Although this positive subjective improvement should not be underestimated, from a critical scientific perspective, it is important to also consider other confounding variables that could underlie such apparently positive effects of neurofeedback interventions. Accordingly, we will focus primarily on biases resulting from repeated measurements (McCall 1985) and discuss the influence of self‐fulfilling prophecies (Jussim 1986), the Hawthorne effect (Sedgwick and Greenwood 2015) and the placebo effect (Kalokairinou et al. 2022).

In the present study we did not use parallel versions of the THI and TFI distress questionnaires, and participants were aware of the aim of the study. Therefore, it is quite conceivable that the participants justified their participation in the study by overestimating some of the subjective benefits. Likewise, it is plausible that the attenuation in tinnitus distress level was caused by the Hawthorne effect, which refers to the tendency of participants to behave differently when they become aware that they are being examined (Sedgwick and Greenwood 2015). In addition, a placebo effect cannot be ruled out, as participants’ expectations regarding neurofeedback may have contributed to perceived improvements independently of actual neural changes (Kalokairinou et al. 2022). In other words, participating in the clinical trial to reduce tinnitus symptoms through neurofeedback may have put participants in a state in which they reported perceived benefits because they thought it was expected of them. Importantly, such behavioral bias is not necessarily incompatible with the fact that we did not detect subjective changes in tinnitus loudness, occurrence, and annoyance over time. Because tinnitus is very irritating and disruptive and cannot be easily suppressed (Langguth et al. 2013), it may have been more difficult for participants to disclose changes in tinnitus‐specific dimensions, especially if their symptoms did not improve at all. For this reason, future studies should attempt to develop subjective screening measures that are not susceptible to such biases in order to provide a stronger argument that neurofeedback can indeed reduce tinnitus distress even in the absence of measurable brain changes in the trained ROIs.

The present study also brought to light a largely unexpected and difficult‐to‐explain finding, namely higher general health scores at T4 compared to T2 in the LI group, as measured by the SF‐36 questionnaire. The SF‐36 questionnaire represents a subjective measurement of functional health and well‐being and is intended to provide a rapid screening of general health status. This instrument includes eight health concepts, namely “physical functioning,” “physical role functioning,” “bodily pain,” “general health perception,” “vitality,” “social role functioning,” “emotional role functioning,” and “mental health” (Lins, 2016). In the present study, we considered it interesting to focus on the “general health perception” subscale as an overall measure to estimate the effect of neurofeedback on health‐related parameters, although we had no clear a priori hypotheses. The reason why an improvement in subjective general health perception was found in the LI but not in the HI group remains unclear and needs to be specifically investigated in future studies. We can only speculate that perhaps one, but not two, neurofeedback sessions per week at the beginning of the study had a positive impact on self‐esteem and lifestyle variables without challenging and overtaxing participants, which may have had a positive impact on overall subjective health assessment scores nonetheless, future studies should attempt to better understand the latent significance of this specific variable and strive to decipher the putative influence of neurofeedback protocols with different training frequencies on more specific health‐related dimensions.

## 5 Limitations

A key limitation of the present study is the lack of available data from the neurofeedback training sessions themselves. As a result, we were unable to directly relate resting‐state EEG changes to participants’ actual neurofeedback performance across the 12 training sessions. It therefore remains possible that the largely null or inconsistent effects observed reflect limited success in up‐ or down‐regulating the targeted neural features. Future studies should include and analyze neurofeedback session data to establish a more direct link between training performance and resting‐state modulation, thereby enabling a clearer interpretation of the observed effects. Another important limitation concerns the interpretation of insula activity. Elevated activation in the insula is frequently reported across a wide range of neurofeedback paradigms and is often linked to general reward processing and learning mechanisms rather than to specific training effects (Sitaram et al. 2017). Therefore, the observed insula activation in the present study should be interpreted with caution, as it may reflect these broader processes rather than changes uniquely attributable to the targeted neurofeedback modulation. A further limitation concerns the restricted analytical scope of the present study. While we did not observe significant changes in resting‐state EEG power within the selected regions of interest, it remains possible that functional connectivity analyses might reveal training‐related alterations at the network level. Such connectivity‐driven changes could occur even in the absence of robust local power effects, reflecting more distributed adaptations in neural communication. Future research should therefore incorporate functional connectivity or whole‐brain analyses to provide a more comprehensive understanding of how neurofeedback training may modulate large‐scale brain dynamics. Moreover, we did not observe significant group differences between the LI and HI training groups. One potential explanation is that the difference in neurofeedback training intensity (one vs. two sessions per week) may not represent a substantial variation in overall training load, particularly given the short duration of individual sessions. Another limitation concerns the manual and approximate adjustment of neurofeedback thresholds by an external operator, which was intended to maintain an 80–90% success rate and ensure participants remained engaged and motivated during training. Unfortunately, data on the applied thresholds were not collected and are therefore unavailable for analysis, limiting the interpretability of individual training success and the conclusions that can be drawn from the present findings.

## 6 Conclusions

The present clinical trial evaluated the effects of multifocal tomographic EEG‐based neurofeedback on neural and psychological dimensions of chronic subjective tinnitus as well as associated health‐related variables. Additionally, we tested the influence of HI and LI neurofeedback on the same outcomes. Our clinical trial demonstrated a positive impact on self‐reported tinnitus distress and overall health parameters, although it is not unlikely that these effects were influenced by procedural biases. Contrary to our hypothesis, we did not detect any resting‐state brain changes in the monitored brain regions, leaving the question open as to whether source‐based neurofeedback in multiple brain regions could have the potential to alter the targeted neural frequency patterns. Given the inherent complexity and extensive parameter space of the neurofeedback approach, as well as the heterogeneity of tinnitus as a targeted disease, which is per se a complex and subjective condition, there are numerous unanswered questions that need to be addressed with rigorous methodology. Therefore, future studies are urgently needed to better assess whether and under what experimental conditions multifocal neurofeedback might provide an advantage over traditional applications in alleviating tinnitus symptoms through functional changes in specific brain areas of interest.

## Author Contributions

S. E. performed formal analysis and wrote the original draft. D. T. conducted data curation, EEG data preprocessing, investigation, and methodology. N. P. contributed to project administration, resources, and supervision. N. G. contributed to review and editing. P. N. contributed to conceptualization, methodology, project administration, supervision, and review and editing. T. K. contributed to project administration and supervision. M. M. contributed to conceptualization, methodology, project administration, funding acquisition, supervision, and writing – review and editing.

## Conflicts of Interest

The authors declare no conflicts of interest.

## Supporting information




**Supplementary Materials**: brb371293‐sup‐0001‐SuppMat.docx

## Data Availability

The individual de‐identified participant data, statistical code and any other materials are available from Martin Meyer on reasonable request.
