# Supplementary material for: EEG‐Based Multifocal Tomographic Neurofeedback in Older Individuals With Chronic Tinnitus Does Not Lead to Persistent Electrophysiological Changes
Source: Brain Behav. 2026 Apr 12;16(4):e71293. doi: 10.1002/brb3.71293 (PMC13071183; doi:10.1002/brb3.71293)
Supplement: Supplementary file 1 — Supplementary Materials: brb371293‐sup‐0001‐SuppMat.docx [file BRB3-16-e71293-s001.docx]

**Consensus on the Reporting and Experimental Design of clinical and cognitive-behavioural Neurofeedback studies (CRED-nf) best practices checklist 2020*** (an online tool to complete this checklist is available at [[rtfin.org/CREDnf](http://www.rtfin.org/CREDnf)](http://www.rtfin.org/CREDnf)).

| **Domain** | **Item #** | **Checklist item** | **Reported on page #** |
| --- | --- | --- | --- |
| **Pre-experiment** | | | |
|  | 1a | Pre-register experimental protocol and planned analyses | N/A |
|  | 1b | Justify sample size | 11 |
| **Control groups** | | | |
|  | 2a | Employ control group(s) or control condition(s) | 11 |
|  | 2b | When leveraging experimental designs where a double-blind is possible, use a double-blind | N/A |
|  | 2c | Blind those who rate the outcomes, and when possible, the statisticians involved | N/A |
|  | 2d | Examine to what extent participants and experimenters remain blinded | N/A |
|  | 2e | In clinical efficacy studies, employ a standard-of-care intervention group as a benchmark for improvement | N/A |
| **Control measures** | | | |
|  | 3a | Collect data on psychosocial factors | 12/13 |
|  | 3b | Report whether participants were provided with a strategy | 11 |
|  | 3c | Report the strategies participants used | N/A |
|  | 3d | Report methods used for online-data processing and artifact correction | 15/16 |
|  | 3e | Report condition and group effects for artifacts | N/A |
| **Feedback specifications** | | | |
|  | 4a | Report how the online-feature extraction was defined | 15/16 |
|  | 4b | Report and justify the reinforcement schedule | 15/16 |
|  | 4c | Report the feedback modality and content | 14-16 |
|  | 4d | Collect and report all brain activity variable(s) and/or contrasts used for feedback, as displayed to experimental participants | 19-30 |
|  | 4e | Report the hardware and software used | 11-20 |
| **Outcome measures** | | | |
| Brain | 5a | Report neurofeedback regulation success based on the feedback signal | 26-30 |
|  | 5b | Plot within-session and between-session regulation blocks of feedback variable(s), as well as pre-to-post resting baselines or contrasts | N/A |
|  | 5c | Statistically compare the experimental condition/group to the control condition(s)/group(s) (not only each group to baseline measures) | 20-30 |
| Behaviour | 6a | Include measures of clinical or behavioural significance, defined a priori, and describe whether they were reached | N/A |
|  | 6b | Run correlational analyses between regulation success and behavioural outcomes | 36-30 |
| **Data storage** | | |  |
|  | 7a | Upload all materials, analysis scripts, code, and raw data used for analyses, as well as final values, to an open access data repository, when feasible | N/A |

*Darker shaded boxes represent *Essential* checklist items; lightly shaded boxes represent *Encouraged* checklist items. We recommend using this checklist in conjunction with the standardized CRED-nf online tool ([rtfin.org/CREDnf](http://www.rtfin.org/CREDnf)) and the CRED-nf article, which explains the motivation behind this checklist and provides details regarding many of the checklist items.
